# AP4M1 as a prognostic biomarker associated with cell proliferation, migration and immune regulation in hepatocellular carcinoma

**DOI:** 10.1186/s12935-023-03089-0

**Published:** 2023-10-11

**Authors:** Yuanhao Peng, Xuanxuan Li, Kuo Kang, Yangying Zhou

**Affiliations:** 1 Department of Oncology, Xiangya Hospital, Central South University, Changsha, 410008 Hunan China; 2 NHC Key Laboratory of Carcinogenesis, Cancer Research Institute, School of Basic Medicine, Central South University, Changsha, 410078 Hunan China; 3 National Clinical Research Center for Geriatric Disorders, Xiangya Hospital, Central South University, Changsha, 410008 Hunan China; 4 Department of General Surgery, Xiangya Hospital Central South University, Changsha, 410008 Hunan China; 5 Hunan Key Laboratory of Precise Diagnosis and Treatment of Gastrointestinal Tumor, Xiangya Hospital Central South University, Changsha, 410008 Hunan China

**Keywords:** AP4M1, Hepatocellular Carcinoma, Clinical prognosis, Immune microenvironment, Biomarker

## Abstract

**Background:**

*AP4M1* is a protein-coding gene that plays a crucial role in transporter activity, recognition, and hereditary-associated diseases, but it’s largely unknown in cancers.

**Methods:**

The expression level of *AP4M1* in cancers was investigated by The Cancer Genome Atlas (TCGA) and Gene Expression Omnibus (GEO) databases, and the correlation between *AP4M1* and hepatocellular carcinoma (HCC) clinicopathological parameters were analyzed. Univariate and multifactorial COX regression analyses were performed to clarify the prognostic value of *AP4M1* in HCC. The correlation between *AP4M1* and immune cell infiltration was analyzed using single-sample Gene Set Enrichment Analysis (ssGSEA). Besides, we verified the biological function of *AP4M1* by applying Cell Counting Kit-8 (CCK8), colony formation, and transwell assays.

**Results:**

The expression of *AP4M1* was significantly elevated in HCC and was correlated with patients’ pathological grades, AFP, and BMI. Kaplan-Meier survival curves indicated that patients with *AP4M1* overexpression had worse overall survival. Univariate and multivariate COX regression analyses showed that *AP4M1* was an independent risk factor affecting the prognosis of HCC. In addition, we observed that *AP4M1* positively correlated with most immune checkpoint suppressor genes in HCC. Moreover, in vitro experiments further confirmed that *AP4M1* could promote the proliferation and invasion of HCC.

**Conclusions:**

*AP4M1* is highly expressed and associated with poor prognosis in HCC. *AP4M1* is closely related to cancer-immune regulation and could be a novel target for HCC, and guiding new strategies for the diagnosis and treatment of HCC patients.

**Supplementary Information:**

The online version contains supplementary material available at 10.1186/s12935-023-03089-0.

## Highlights

1. The expression of *AP4M1* was significantly elevated in HCC and was significantly correlated with patients’ pathological grade, AFP, and BMI.
2. *AP4M1* was associated with unfavorable prognosis in HCC, and was an independent risk factor for HCC prognosis.
3. *AP4M1* positively correlated with most immune checkpoint suppressor genes in HCC and could be a novel immunotherapy target for HCC.
4. *AP4M1* may be involved in the malignant progression of HCC, as well as the cancer immune regulation.

**Supplementary Information:**

The online version contains supplementary material available at 10.1186/s12935-023-03089-0.

## Introduction

Globally, liver cancer has an increasing incidence and mortality, which poses a severe threat to human health and the economy [1, 2]. Hepatocellular carcinoma (HCC) is the most common type of primary liver cancer, accounting for approximately 80% of all cases [3]. According to statistics, there are more than 900,000 new diagnoses and more than 800,000 deaths each year [4]. In the past decade, even though great progress has been made in the treatment and diagnosis of hepatocellular carcinoma, the overall survival rate of patients is still low [5]. Since the majority of patients with HCC were diagnosed in the advanced stages, or with invasion and metastasis within and outside the liver, the optimal time for surgical treatment was lost [6, 7]. The oncogenesis of HCC is considered to be a complex multifactorial process, and the biological and clinical diversity of HCC presents a great challenge for individualized clinical treatment. Therefore, exploring biomarkers of HCC is crucial to improve early diagnosis and finding therapeutic targets.

*AP4M1* is a component of the adaptor protein complex 4 and is involved in the coding of the adaptor protein complex 4, also known as SPG50. AP-4 compounds have been involved in trafficking of transmembrane proteins from the trans-Golgi network to early and late endosome [8, 9]. The *AP4M1* gene is highly expressed in the brain, especially during fetal development [10]. Interruptions in *AP4M1*’s ability to affect its function can impair normal brain development and may impair the excitability of neurons. Studies have shown that *AP4M1* is involved in the pathological process of congenital anthropogenic paralysis, suggesting that it may be damaged by analogous glucose-mediated proteins through the early neural axis and sequential white loss [11].

At present, there is no relevant studies report on *AP4M1* in cancers. It has been reported that the autophagy protein ATG9A is a product of AP-4, and that deletion of AP-4 leads to mislocalization of *ATG9A*, which may affect the transport and function of *ATG9A* in axons [12–14]. Given the close relationship between autophagy regulation and tumorigenesis, the evidence for the role and clinical significance of *AP4M1* in the diagnosis, disease progression, and prognosis of HCC is insufficient. Therefore, this study proposed to investigate the expression of *AP4M1* in HCC and its role in HCC development and prognosis.

In this study, we conducted a comprehensive analysis using clinical characteristics and survival data of HCC in a public database to assess the significance of *AP4M1*expression in HCC. We found that the high expression of *AP4M1* was related to the inferior prognosis and cancer-immune regulation in HCC. The upregulated *AP4M1* also accelerated the proliferation and invasion ability of HCC. Thus, our research identified the potential role of *AP4M1* in the onset and development of HCC, and could be a novel diagnostic and prognostic biomarker.

## Materials & methods

### Data Collection

The expression data of *AP4M1* in pan-cancer were obtained from the Cancer Genome Atlas (TCGA) database, and the RNAseq data of patients with hepatocellular carcinoma in the TCGA-LIHC dataset were extracted. The formatted RNAseq data were converted to TPM format, and the clinical data of 424 patients with hepatocellular carcinoma were obtained for subsequent analysis after removing the patients without clinical information.

### Comparison of the expression differences of *AP4M1* in HCC and normal tissues

Firstly, the expression of *AP4M1* in the different types of cancer tissue including HCC tissues was analyzed through the Xiantao tools. Then, the Biomarker Exploration of Solid Tumors (BEST, https://rookieutopia.com/app_direct/BEST/) network tool was used to compare the expression of *AP4M1* in hepatocellular carcinoma and normal tissues in GSE144269, GSE14520, GSE54236 and TCGA-LIHC datasets. The gene expression levels were transformed into a Z score. Also, AP4M1 protein expression in HCC and normal tissues were obtained from the CPTAC database, a proteomic database that includes a variety of cancers and enables users to obtain proteomic and genomic information on a large scale [15, 16].

### Analysis of the association of *AP4M1* and HCC clinicopathological parameters

After analyzing the protein and mRNA expression levels of *AP4M1* in HCC, the TCGA-LIHC dataset was utilized to assess the clinical relevance of pathological parameters for hepatocellular carcinoma. In this study, we carried out the normality test for the numerical type of variables and used the expression median to categorize the groups. When the data meet the normal distribution, we will calculate the mean ± standard deviation (SD) with the Z-score transform of the corresponding variables; If it does not conform to the normal distribution, the median of the related variables (upper quartile and lower quartile) will be calculated [17, 18].

### Survival analysis

To investigate the prognostic impact of *AP4M1* mRNA on HCC samples. We first analyzed the effects of *AP4M1* on overall survival (OS), recurrence-free survival (RFS), progression-free survival (PFS) and disease-specific survival (DSS) in hepatocellular carcinoma patients in the Kaplan-Meier (KM) plotter website (http://www.kmplot.com/analysis/) [19]. In addition, hazard ratios (HRs) and log-rank p-values with 95% confidence intervals (CI) were determined [20]. Subsequently, HCC patients were divided into high and low groups according to *AP4M1* expression levels, and proportional risk hypothesis tests were performed using “survival” packages and the prognostic value of *AP4M1* on overall survival in HCC was assessed by univariate and multifactorial Cox regression analysis.

### Analysis of *AP4M1* gene alternations in HCC

The cBioPortal database (version 3.7.1, http://cbioportal.org) was primarily used to investigate multivariate cancer genomics datasets containing resources from 20 cancer studies and more than 5,000 tumor samples [21, 22]. A TCGA-LIHC (Firehose Legacy) dataset containing 379 samples was selected, and normalized RNA Seq V2 RSEM data was used for mutation analysis.

### Correlations between *AP4M1* and the immune environment

The relationship between *AP4M1* expression and immune cell infiltration was analyzed by the ssGSEA method using Xiantao Tools and presented with a lollipop plot. ssGSEA calculated the number of immune cells in tumor specimens based on gene expression data and the R package (gsva.20), and then used Spearman rank correlation analysis to determine the relevance of *AP4M1* and 24 immune cell infiltration level and used the ggplot2 package for visualization. The Tumor Immune Evaluation Resource (TIMER, https://cistrome.shinyapps.io/TIMER/) is used to analyze immune infiltration in different types of cancer [23]. We explored the relationship between the altered somatic copy number of the *AP4M1* gene and infiltrating immune cells in HCC by using the SCNA module. A cut-off value of P < 0.05 was used. The TISIDB database was used to further analyze the expression of *AP4M1* in immune subtypes of liver cancer [24].

### *AP4M1* co-expression gene analysis and gene enrichment analysis

The LinkedOmics database (http://www.linkedomics.org/) is a combined multi-omics dataset from the CPTAC and TCGA databases, including clinical data with 32 cancer types and mass spectrometry-based proteomics data [25]. We used the LinkedOmics database for *AP4M1* co-expression gene analysis. The top 50 genes positively and negatively associated with *AP4M1* in LIHC were obtained through the LinkFinder module. The *AP4M1* gene set was enriched for analysis in the LinkInterpreter module and 500 simulations were performed.

### Cell culture, antibodies, siRNA and plasmids

The human liver cancer and normal liver cell lines were acquired from American Type Culture Collection (ATCC). All cells were maintained in Dulbecco’s Modified Eagle’s Medium (DMEM, Life Technologies) with 10% fetal bovine serum (FBS). Cells were cultured in a humidified incubator at 37 °C and in an atmosphere of 5% CO_2_. All the cell lines tested negative for mycoplasma contamination. Additionally, prior to their use, all cell lines underwent authentication through short tandem repeat profiling. Furthermore, these cell lines were passaged fewer than ten times after being initially revived from frozen stocks. The primary antibodies were Beta Actin Monoclonal antibody (Cat# 60318-1-lg; Proteintech, Wuhan, China; 1:10000), and *AP4M1* Polyclonal antibody (Cat# 11653-1-AP; Proteintech, Wuhan, China; 1:500). The siRNA of *AP4M1* were purchased from RibBio (RibBio, Guangzhou, China). HanBio designed and established the *AP4M1* overexpression plasmid (HanBio, Shanghai, China). Plasmid and siRNA transfection was performed with Lipofectamine® 3000 following the manufacturer’s instructions.

### Western blot analysis

The Western blot analysis was performed as we previously described [26]. The difference is that quantity of 20 µg of total protein was used for western blot analysis. Primary antibodies against *AP4M1* and Beta Actin were purchased from Proteintech (Proteintech, Wuhan, China).

### Cell proliferation assay

Cells were seeded at a density of 1 × 10^3^ cells/well in DMEM medium (100 µl) into 96 well plates. Each group had five replicate wells after 24, 48, 72 and 96 h, cell viability was determined by the Cell Counting Kit-8 (CCK8) method. Adding 10 µl CCK8 solution to each well (be careful not to create bubbles) and put the culture plate in the incubator and incubating for 2 h. The absorbance at 450 nm was measured with an enzyme label and a cell proliferation curve was plotted.

### Plate-colony formation assay

Cells (500/well) were seeded into 6-well plates and cultured in 3 ml DMEM supplemented with 10% FBS for about 2 weeks, changing the culture medium every five days during the period. After the colony grew, it was fixed with methanol and stained with 0.1% crystal violet for 30 min, then scored using a microscope and Image J software.

### Cell migration and invasion assays

Cell migration assays : Add 200 µl of DMEM without FBS into the transwells and incubate for 30 min. We then add 4 × 10^4^/well cells and 200 µl DMEM without FBS in the upper layer and 800 µl DMEM with 10% FBS in the lower layer of the transwell. Put the culture plates in the incubator and incubate for 24 h. Then wipe the cells in the upper transwell, and fix them with 10% methanol for 10 min and stain them with 0.1% crystal violet for 30 min.

Cell invasion assays: Add 70 µl of 10% matrigel matrix onto the upper chamber and place it in the cell incubator for solidification. Then add 200 µl of DMEM without FBS to the transwells and incubate for 30 min. Next, add 1 × 10^5^ cells in 200 µl of DMEM without FBS to the upper layer, and 800 µl of DMEM with 10% FBS to the lower layer of the transwell. Place the culture plates in the incubator and incubate for 48 h. Afterward, remove the cells in the upper transwell, fix them with methanol for 10 min, and stain with 0.1% crystal violet for 30 min.

## Results

### *AP4M1* expression is elevated in HCC

**Fig. 1:**
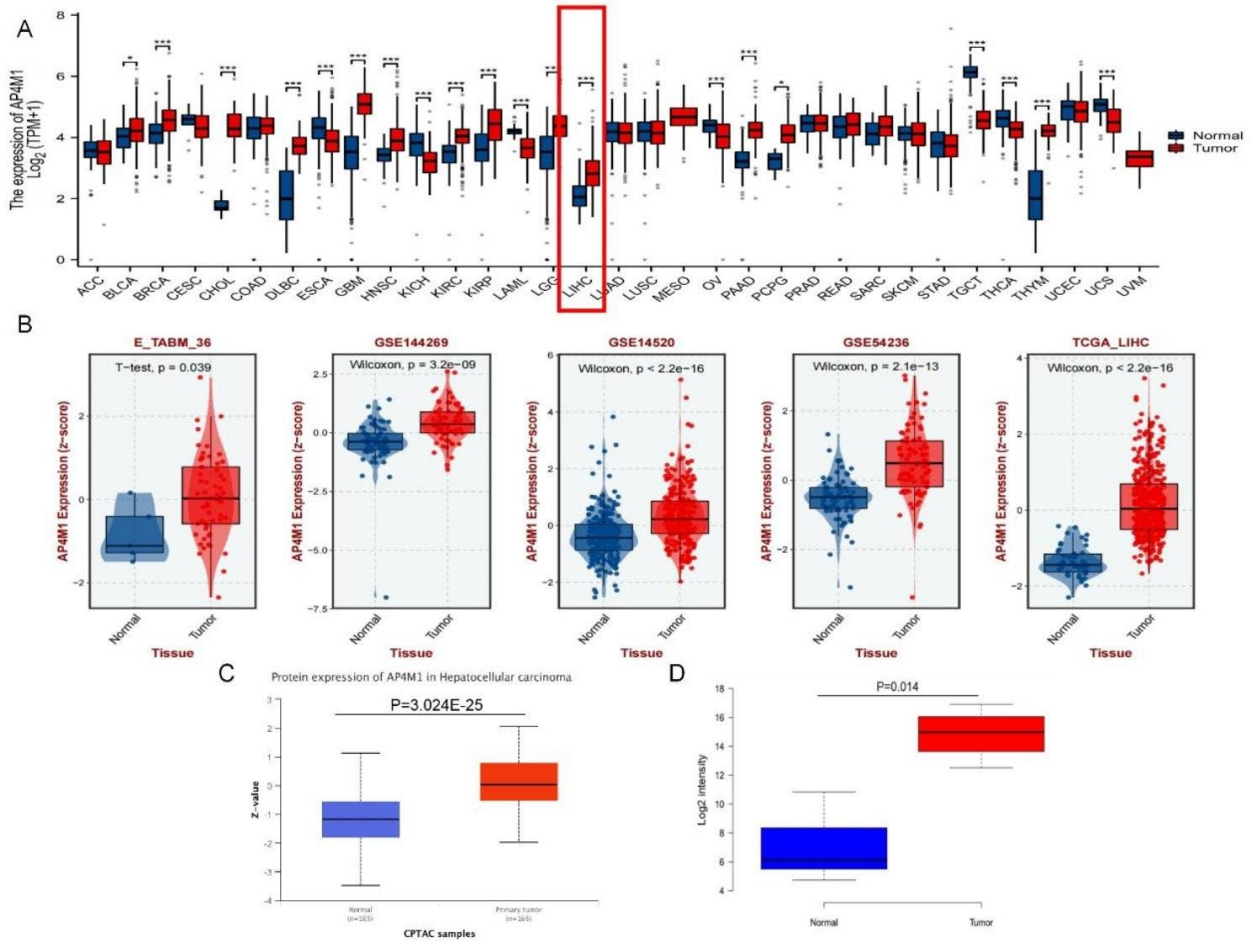
***AP4M1*is highly expressed in liver cancer**. (**A**) *AP4M1* expression in various tumors in TCGA dataset. (**B**) The GEO dataset and TCGA-LIHC dataset download from the BEST database showed that AP4M1 was highly expressed in HCC. (**C**) AP4M1 protein expression levels obtained from CPTAC database. (**D**) AP4M1 protein levels from the Pan-cancer proteomics study

To explore the potential role of *AP4M1* in HCC, we first analyzed the expression data from various databases. By evaluating the expression of *AP4M1* in each type of cancer in the TCGA database, we discovered that the levels of *AP4M1* mRNA are significantly elevated in HCC (Fig. 1A). The analysis of the GSE144269, GSE1520 and GSE54236 datasets from the BEST database also confirmed that *AP4M1* expressions in HCC tissues were significantly higher than in normal (Fig. 1B). Furthermore, we analyzed the AP4M1 protein levels from the Pan-cancer proteomics study [27] and CPTAC database, and the results were in line with our finding that the AP4M1 protein levels were increased in HCC (Fig. 1C-D). These results suggested that *AP4M1* overexpression may play an essential role in the development of HCCs. Besides, we discovered that *AP4M1* was accumulated below the 0.963 ROC curve for distinguishing HCC from normal tissue (Supplementary Fig. 1A). We further compared AP4M1 with three other biomarkers. Our result showed that the AUC values of the ROC curves using DDK-1, AXAN2 and GPC3 to distinguish tumor tissue from normal tissue were 0.749, 0.895 and 0.919, respectively (Supplementary Fig. 1B-D). Taken together, *AP4M1* presented an excellent performance in the diagnosis of HCC patients.

### Analysis of correlation of *AP4M1* expression levels with clinical pathological characteristics of HCC

**Fig. 2:**
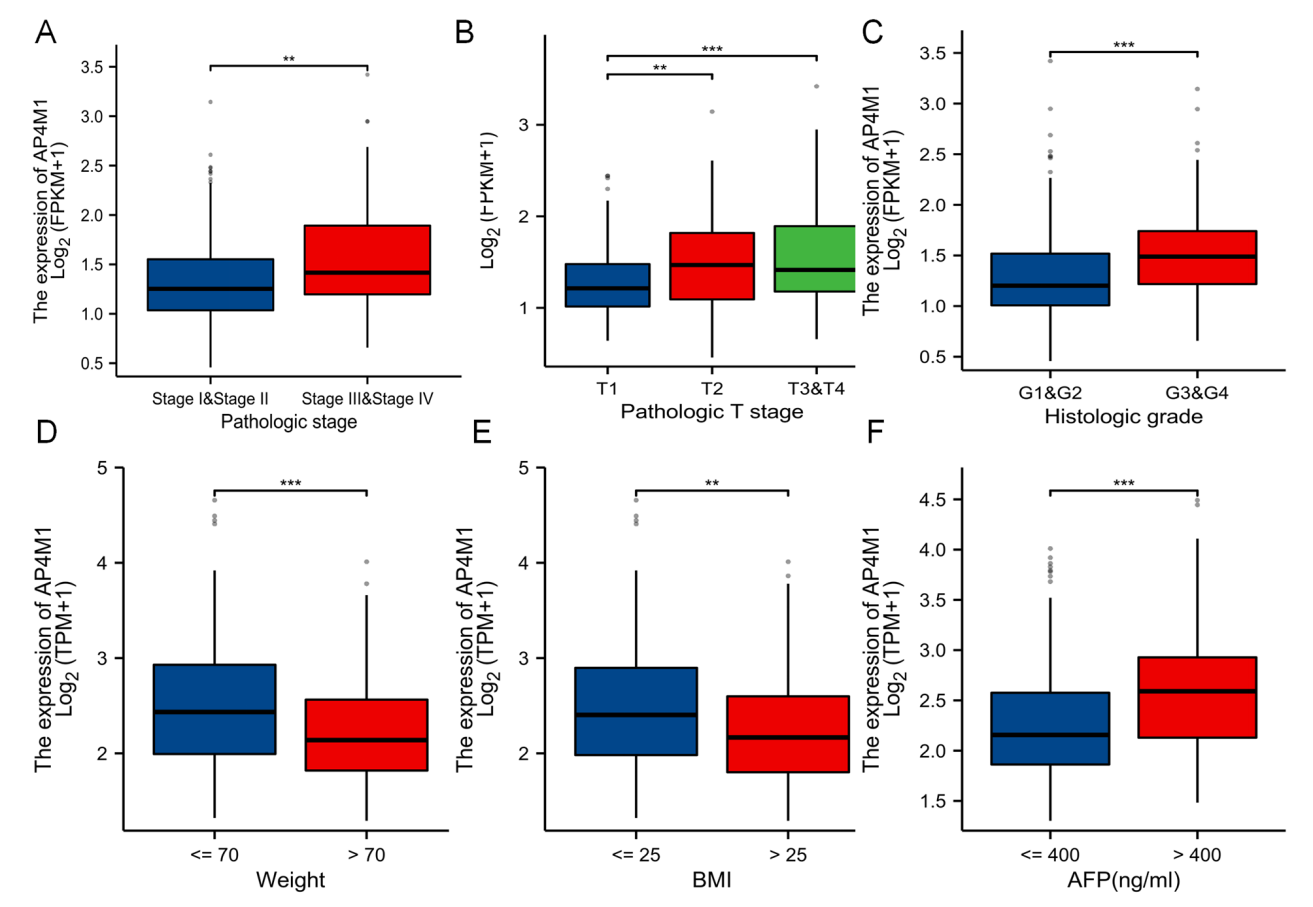
**The expression of*AP4M1*in different clinicopathologic features of liver cancer**. (**A**) Pathologic stage. (**B**) Pathologic T stage. (**C**) Histologic grade. (**D**) Weight. (**E**) BMI. (**F**A) FP. (* *p* < 0.05; ** *p* < 0.01; *** *p* < 0.001, ns, no significance)

We further explored the correlation of the *AP4M1* with the clinical characteristics of HCC, as well as the role of the *AP4M1* in prognosis in HCC. Firstly, based on the TCGA-LIHC dataset, the relationship between clinical pathological characteristics of HCC patients and levels of expression of *AP4M1* were presented in Supplementary Table 1. The results showed that patients with high and low expression of *AP4M1* in HCC had significant differences between pathologic stage, pathological T stage, histologic grade, histologic type, weight, BMI, AFP, and OS. As illustrated in Fig. 2, the trend toward increased *AP4M1* expression with advanced pathologic stage, T stage, or histologic grade was observed (Fig. 2A-C). Decreased expression of *AP4M1* was found in patients with body weight > 70 kg and BMI > 25 (Fig. 2D-E). We also explored that the mRNA expression levels of *AP4M1* in liver cancer patients at AFP > 400 were significantly higher than in the AFP ≤ 400ng/ml group (Fig. 2F). Besides, there is no significant difference in *AP4M1* expression according to age, N and M stage.

### *AP4M1* high expression related to the poor prognosis in HCC

**Fig. 3:**
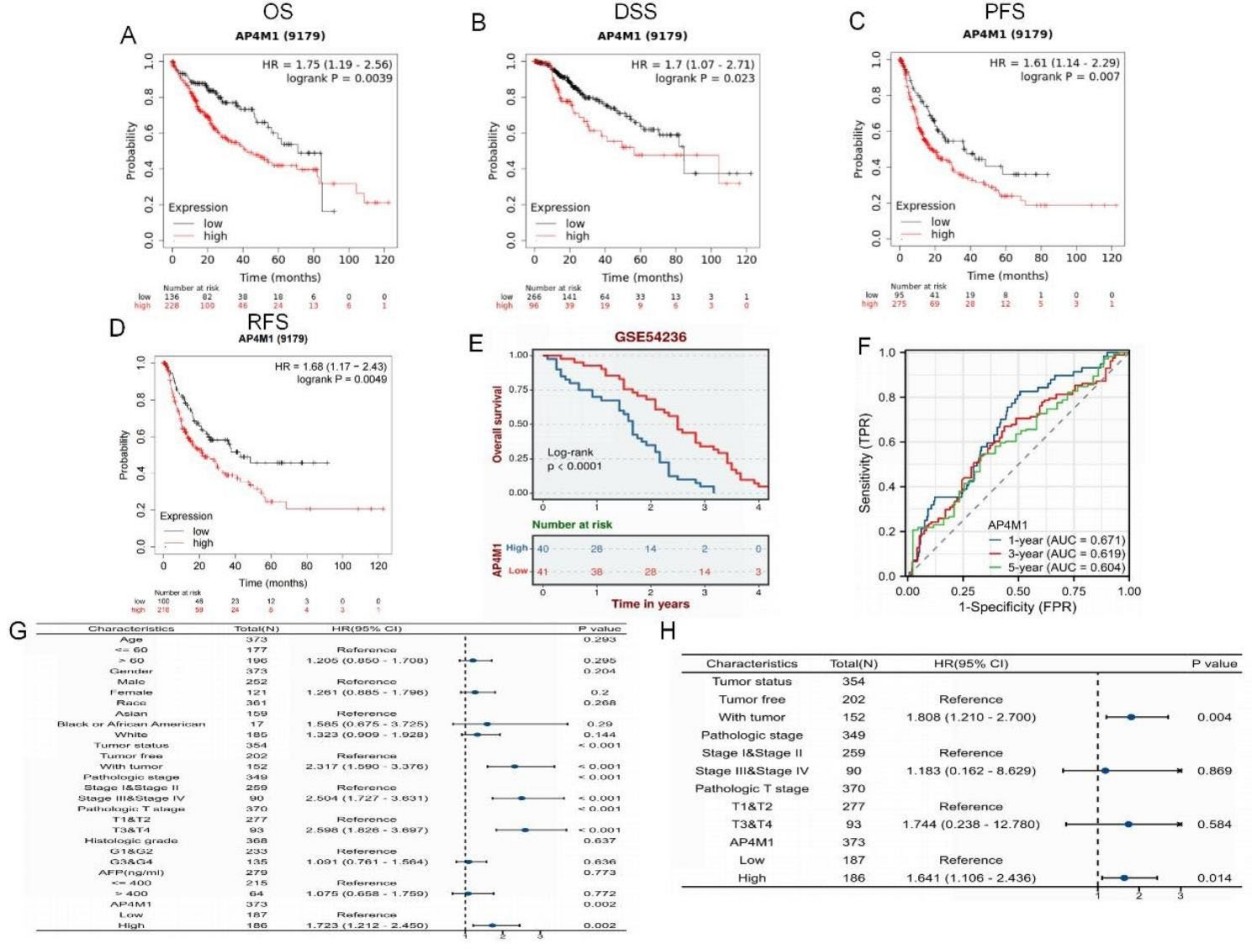
**Prognostic value of*AP4M1*in HCC**. (**A**) Overall survival analysis of *AP4M1* mRNA high and low expression in HCC. (**B**) DSS analysis of *AP4M1* mRNA high and low expression in HCC (**C**)PFS analysis of *AP4M1* mRNA high and low expression in HCC (**D**) RFS analysis of *AP4M1* mRNA high and low expression in HCC. (**E**) Survival curves of high and low AP4M1 expression in the GSE54236. (**F**) Time-dependent ROC curve. (**G**) Univariate and (**H**) multivariate COX regression analysis of OS correlation in HCC. OS: overall survival; PFS: Progression Free Survival; RFS: Recurrence Free Survival; DSS: Disease Specific Survival; HCC: hepatocellular carcinoma

Using KM-plotter and BEST databases, the survival curve of *AP4M1* was initially generated, and the results indicated that elevated expression of *AP4M1* was associated with a poor prognosis for the HCC patient. OS, DSS, PFS, and RFS were significantly lower in the high-expression group of *AP4M1* compared to the low-expression group (Fig. 3A-E). The results of the KM curve suggested that *AP4M1* can be used as an indicator of the progression and prognosis of HCC patients. The time-dependent ROC curve analysis showed that the area under the curve (AUC) values for the predicted 1-, 3-, and 5-year survival rates of HCC patients based on the *AP4M1* expression levels were above 0.6 (Fig. 3F).

In order to determine the risk factors related to HCC survival, we further used both univariate and multivariate Cox regression analysis. Univariate Cox analysis indicated that *AP4M1* (HR = 1.732, *p* = 0.002), tumor status (HR = 2.317, *p* < 0.001), pathologic stage (HR = 2.504, *p* < 0.001) and pathologic T stage (HR = 2.598, *p* < 0.001) were associated with patients’ OS (Fig. 3G). We further conducted multivariate Cox regression analysis and depicted as a forest boxplot in Fig. 3H, which demonstrated that tumor status (HR = 1.808, *p* = 0.004) and *AP4M1*(HR = 1.641, *p* = 0.014) were independent predictors of HCCprognosis, implying a crucial role of *AP4M1* in HCC.

### Genetic alteration analysis of *AP4M1*

**Fig. 4:**
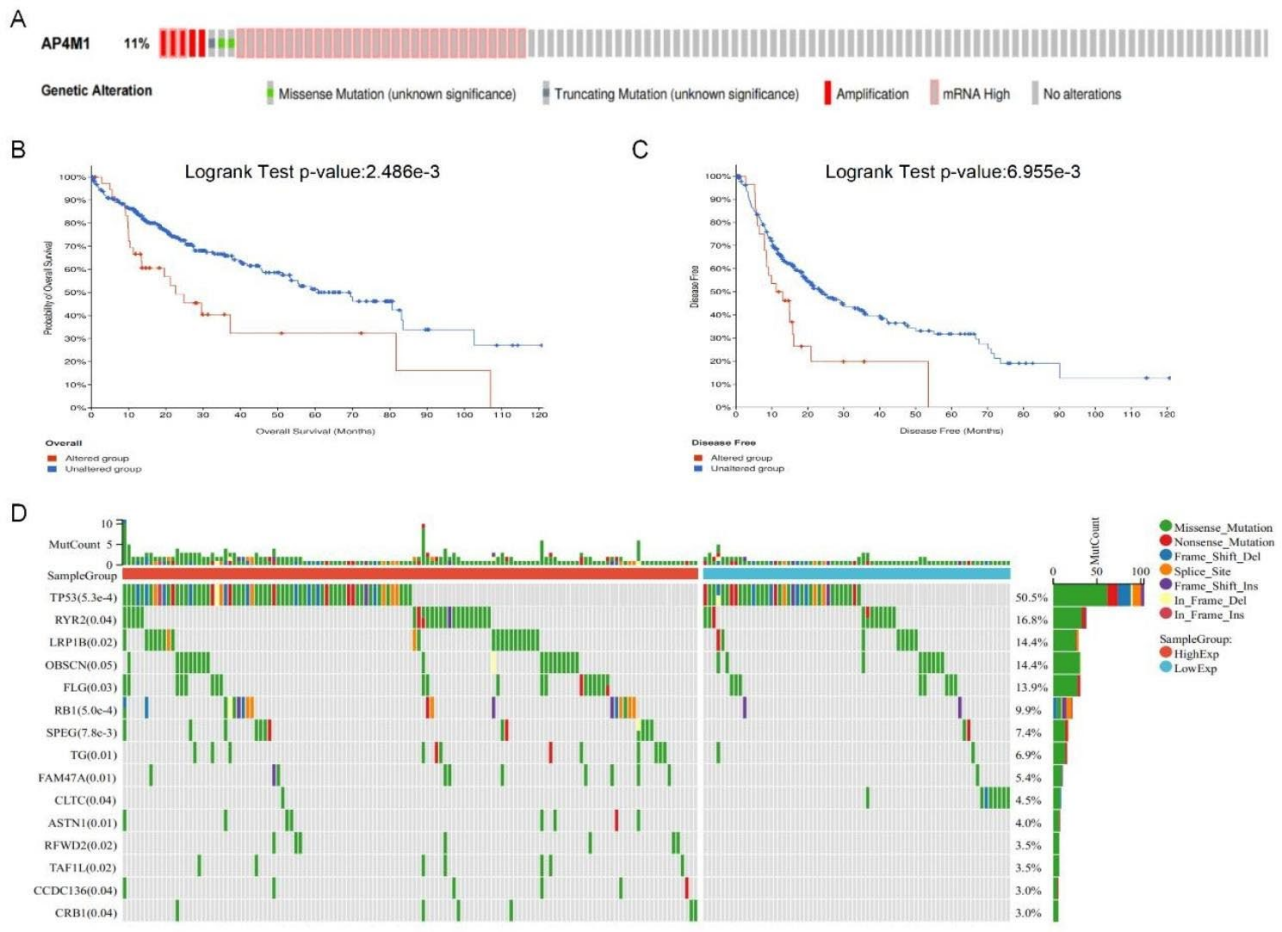
**AP4M1 genetic alterations in HCC**. (**A**) Types of *AP4M1* mutation in HCC. (**B**) *AP4M1* alteration was associated with overall survival in HCC. (**C**) *AP4M1* alteration was associated with disease-free survival in HCC. (**D**) Somatic landscape of HCC in *AP4M1*-high and *AP4M1*-low subgroup

Considering that genomic changes in *AP4M1* are also vital, we used the cBioPortal database to study the amplification frequency and genetic change types of the *AP4M1* in HCC. The frequency of gene alternation of *AP4M1* in HCC was 11%, including missense mutation, truncating mutation, amplification and high mRNA (Fig. 4A). We further analyzed the relationship between *AP4M1* gene alterations and HCC prognosis, and Kaplan-Meier plots and log-rank tests showed significant differences in OS (*p* = 2.486 × 10^-3) (Fig. 4B) and DFS (*p* = 6.955 × 10^-3) (Fig. 4C) between patients with and without gene alterations. Figure 4D summarized and somatic mutation landscape in *AP4M1* high and low expression groups in HCC samples. The waterfall plot illustrated the top 15 most commonly mutated genes. Among them, *TP53* ranked the most mutated gene with a more than 50% mutation rate.

### Correlation analysis of *AP4M1* with immune cell infiltration in HCC

**Fig. 5:**
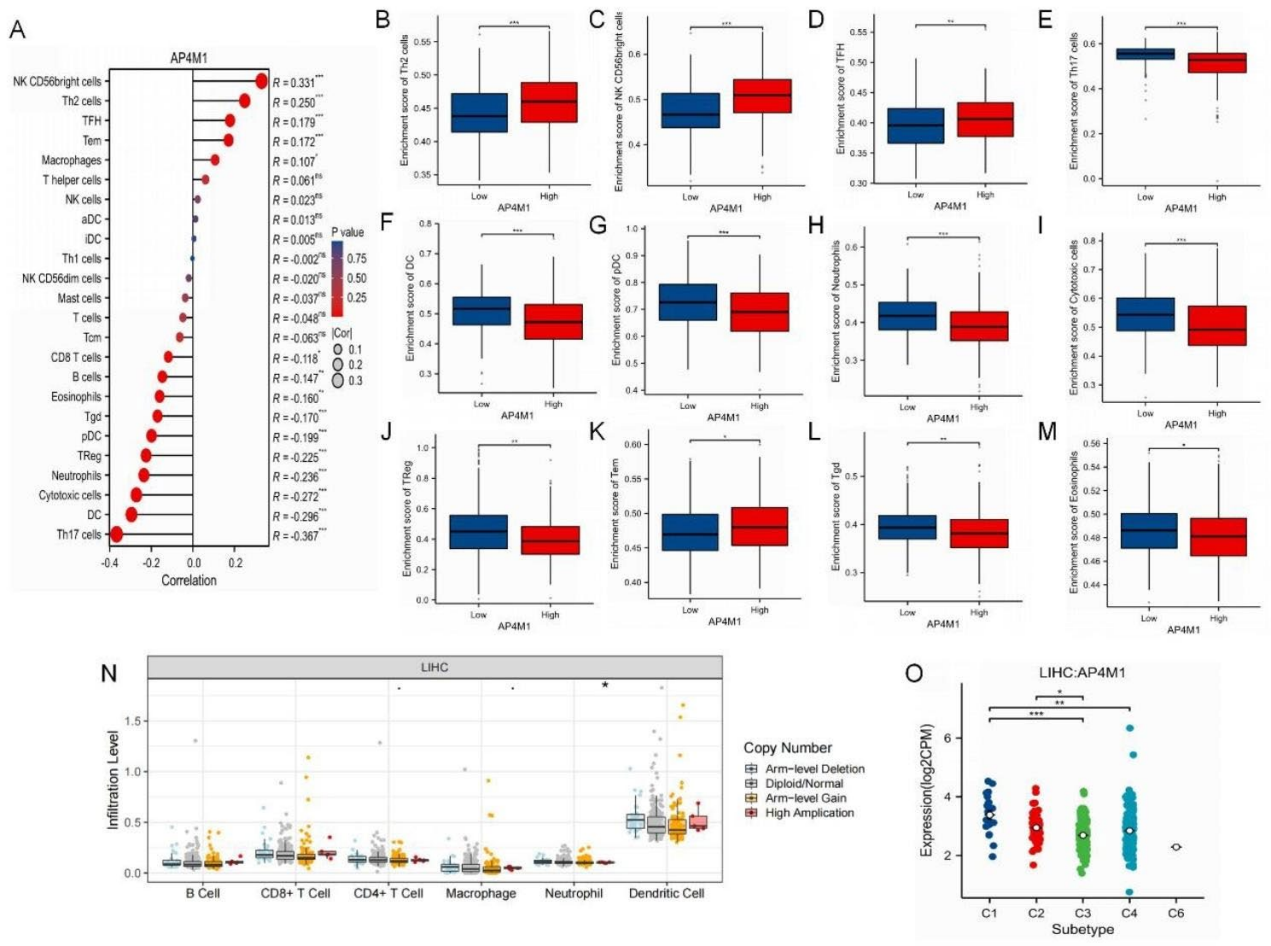
**Association between*AP4M1*and immune cell infiltration in HCC**. (**A**) *AP4M1* is correlated with immune infiltration in HCC. (**B-M**) According to different expression levels of *AP4M1*, the infiltration levels of immune cells were analyzed in groups. (**N**) The relationship between the altered somatic copy number of *AP4M1* gene and infiltrating immune cells in HCC. (**O**) The expression of *AP4M1* in immune subtypes of liver cancer. (* *p* < 0.05; ** *p* < 0.01; *** *p* < 0.001, ns, no significance)

Using the ssGSEA method, we validated and quantified the associations between *AP4M1* expression and immune cell infiltration levels. The expression of *AP4M1* was positive with NK CD56bright cells, TH2 cells TFH, Tem and Marcophages but negative with Th17 cells, DC, Neutrophils, cytotoxic cells, Treg, Tcm, pDC, CD8^+^ T cell and B cell (Fig. 5A). Subsequently, HCC samples were divided into *AP4M1*-high and *AP4M1*-low expression groups, and we sought to determine whether various expression groups of *AP4M1* differ in the HCC tumor immune microenvironment. In the *AP4M1* high-expression group, Th2, CD56bright cells and TFH were found to be elevated, while Th17 cells, DC, pDC, neutrophils, cytotoxic cells, Treg, Tem, Tgd, eosinophils, CD8 + T cell and B cell expressions were decreased (Fig. 5B-M). These results implied that *AP4M1* high group may have lower anti-cancer immune ability than *AP4M1* low group. The TIMER database was utilized to determine whether *AP4M1* expression in HCC was connected with immune cell invasion levels. The results indicated that the CNV of *AP4M1* was related to the level of neutrophil infiltration (Fig. 5N). The TISIDB database was then used to investigate the role of *AP4M1* expression in the immunological subtypes and molecular subtypes of HCC. The results indicated that the expression of *AP4M1* within HCC was connected with various immunological subtypes and molecular subtypes, and with the C1 subtype having the highest expression (Fig. 5O).

### The correlation between immunotherapy and *AP4M1* expression in HCC

**Fig. 6:**
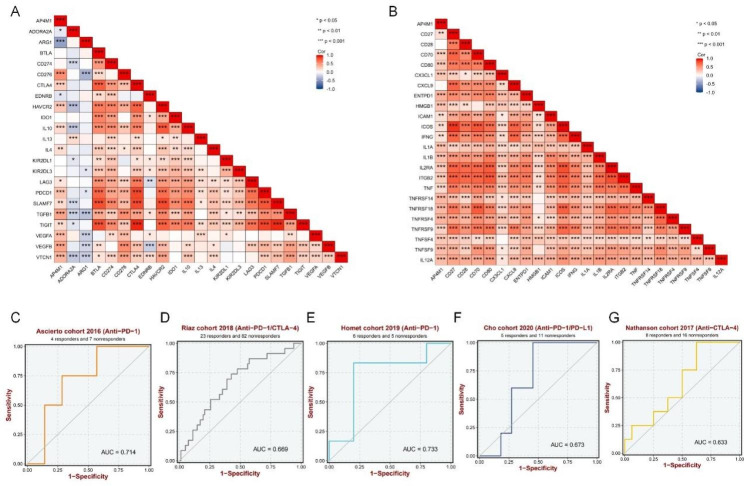
**The correlation analysis of*AP4M1*expression and immune checkpoint genes**. (**A**) Immunostimulatory factors (**B**)Immunosuppressive factors. (**C-G**) Diagnostic value of *AP4M1* for differentiating immunotherapy responses in an immunotherapy cohort. (* *p* < 0.05; ** *p* < 0.01; *** *p* < 0.001, ns, no significance)

The association of high *AP4M1* expression with immunotherapy tolerance in HCC was further examined. First, we explored the correlation between *AP4M1* and immune checkpoint-related genes. We found that most immune checkpoint inhibitors, such as *CTLA4, HAVCR2, LAG3, TGFB1, TIGIT* were significantly negatively correlated with *AP4M1* (Fig. 6A). Furthermore, we observed that AP4M1 was positively correlated with immune checkpoint stimulators except for *CD28* and *CXCL9* (Fig. 6B). In addition, *AP4M1* was evaluated for its ability to differentiate immune responses in an immunotherapy cohort. We discovered that the area under the receiver operating characteristic curve (AUC) values were 0.714, 0.669, 0.733, 0.673 and 0.633 in the Ascierto, Riaz, Homet, Cho and Nathanson cohorts, respectively, indicating that *AP4M1* had a good performance in distinguishing anti-PD-1/PD-L1 respondents and non-responders (Fig. 6C-G).

### Functional enrichment analysis of *AP4M1* in HCC

**Fig. 7:**
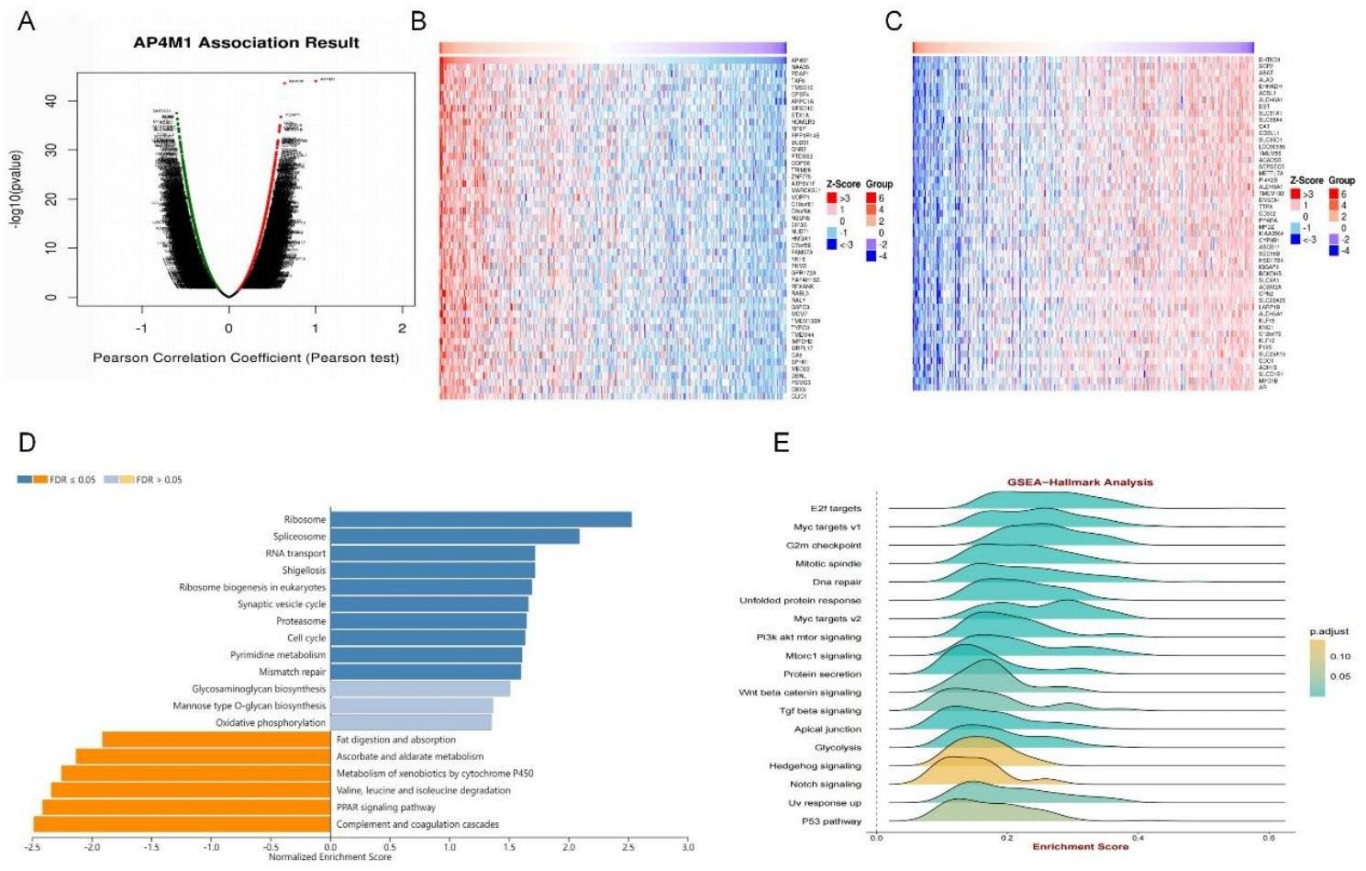
**Co-expressed genes of*AP4M1*in HCC and gene functional analysis of*AP4M1*in HCC**. (**A**) *AP4M1* co-expressed gene volcano map obtained from Linkedmoics. (**B-C**) Heat maps of the expression of the top 50 associated genes that were positively and negatively correlated with *AP4M1*. (**D**) KEGG analysis. (**E**) GSEA-Hallmark signaling pathway enrichment analysis

To further investigate the possible role of *AP4M1* in HCC, we explored the co-expression genes of *AP4M1*. As illustrated in Fig. 7A-C, the top 50 genes that were significantly negatively and positively correlated with *AP4M1* were acquired from the Linkedomics database. These co-expressed genes were further analyzed in GO and KEGG enrichment analysis to clarify the role of *AP4M1* in HCC. The results of GO and KEGG analyses showed that the most important biological processes (BP) of *AP4M1* included translational initiation, RNA splicing, DNA conformation change, cell cycle G2/M phase transition and ephrin receptor signaling pathway (Supplementary Fig. 2A). The most enriched cellular components (CC) were Sm-like protein family complex, cytosolic part, small nucleolar ribonucleoprotein complex, heterochromatin and dendritic shaft (Supplementary Fig. 2B). The most enriched molecular functions (MF) were rRNA binding, structural constituent of cytoskeleton, serine hydrolase activity, cell adhesion molecule binding (Supplementary Fig. 2C). KEGG enrichment results suggested that *AP4M1* and co-expressed genes were mainly involved in the Ribosome, Spliceosome, RNA transport, Shigellosis, Ribosome biogenesis in eukaryotes, Synaptic vesicle cycle, Proteasome, Cell cycle, Pyrimidine metabolism, Mismatch repair (Fig. 7D). Based on the results of the GSEA-Hallmark signaling pathway enrichment analysis, we found *AP4M1*-related genes were mainly concentrated in E2f targets, Myc targets V1 and G2M checkpoint, etc. (Fig. 7E).

### Validation of the biological function of *AP4M1* in HCC

**Fig. 8:**
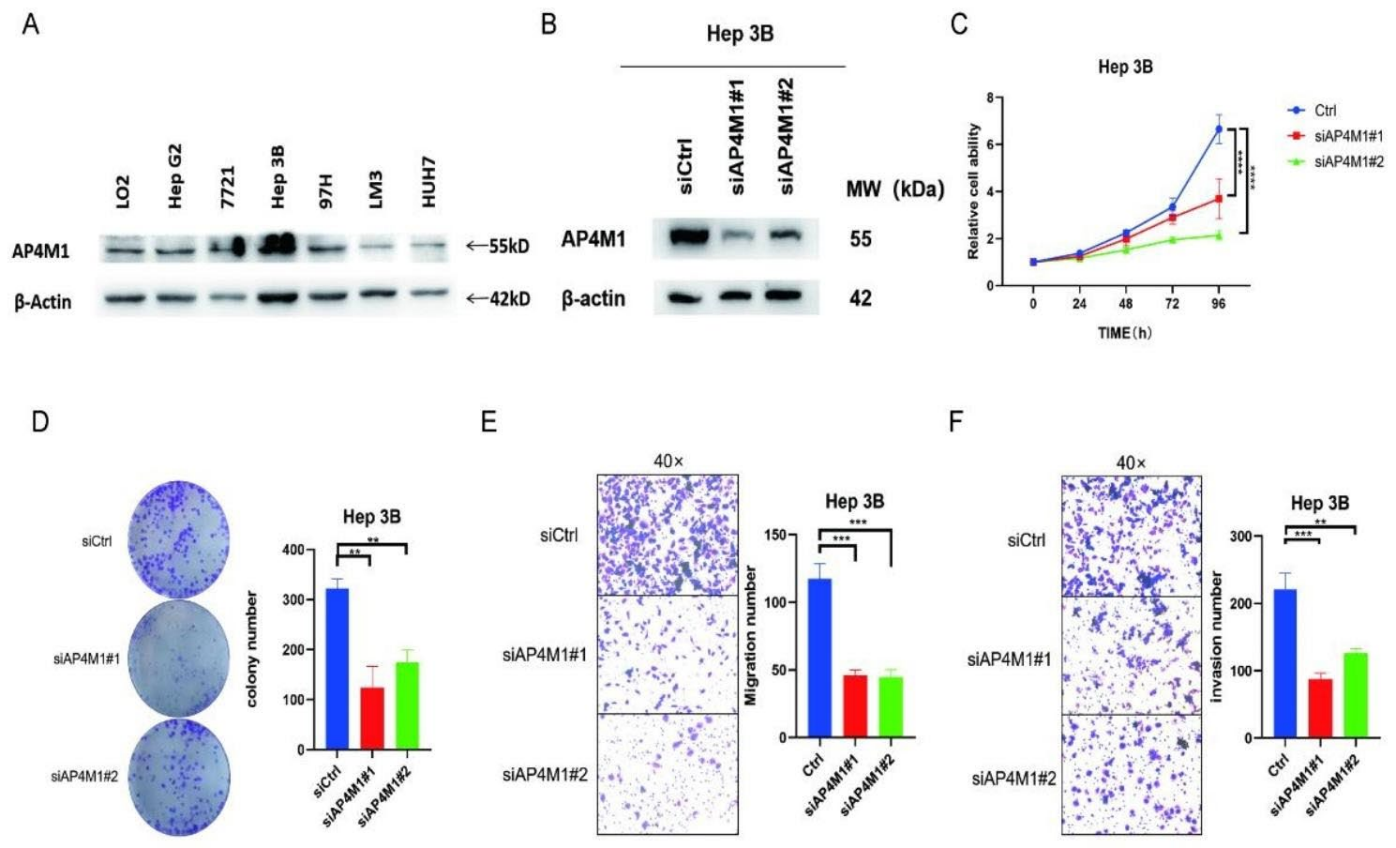
**Depletion of AP4M1 inhibits the proliferation, migration and invasion of HCC**. (**A**) The expression of *AP4M1* in the HCC cell lines were detected by western blotting. (**B**)The transfection efficiency of si-*AP4M1* in Hep 3B. (**C**) The effect of *AP4M1* knockdown on cell proliferation was detected by CCK8 assay. (**D**) Colony formation assay showed *AP4M1* knockdown group were significantly less than siCtrl group cells. (**E-F**) Transwell assay showed migration (**E**) and invasion ability (**F**) after *AP4M1* depletion

**Fig. 9:**
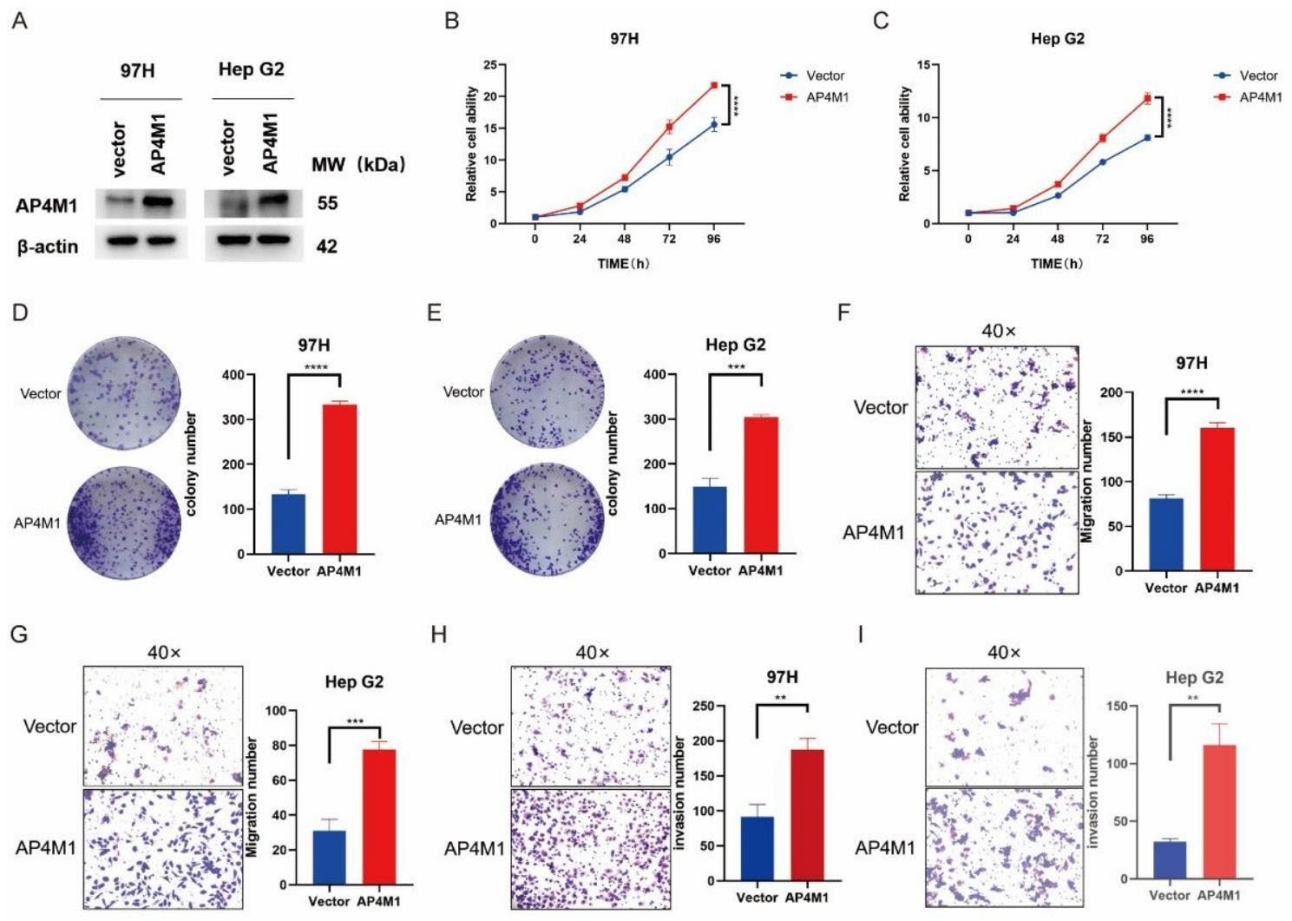
**Overexpression of*AP4M1*can promote the proliferation, invasion and migration of HCC**. (**A**) *AP4M1* was overexpressed in 97 H and HepG2 cell lines (**B-C**) The effect of *AP4M1* overexpression on cell proliferation was detected by CCK8 assay. (**D-E**) Colony formation assay. (**F-I**) Transwell assay shows migration (**F**-**G**) and invasion ability (**H**-**I**) after *AP4M1* overexpression

To verify the role of *AP4M1* in the development of HCC cells, we first detected the AP4M1 expression in six different HCC cell lines (Fig. 8A). We selected Hep3B cell line with higher expression levels of *AP4M1* as the experimental cell line. Western blot assay presented the transfection effectiveness of *AP4M1* siRNA in Hep3B cells (Fig. 8B). CCK8 assay demonstrated that the depletion of *AP4M1* decreased the cell proliferation rate dramatically (Fig. 8C). Colony formation assay also presented that the knockdown of *AP4M1* corresponded to a reduction in clonogenicity (Fig. 8D). Consistently, the transwell assays showed that the depletion of AP4M1 inhibited both cell migration and invasion capacity obviously (Fig. 8E-F). In addition, we overexpressed *AP4M1* in 97 H and HepG2 cells (Fig. 9A). The results of the CCK8 assay showed that the overexpression of *AP4M1* increased the cell proliferation rate dramatically, and the results from the colony formation assay presented that compared with the vector group, the overexpressed *AP4M1* increased the clonogenicity of HCC cells (Fig. 9B-E). Moreover, the transwell assay revealed increased cell invasion and migration capacity when *AP4M1* was overexpressed (Fig. 9F-I). The above results indicate that *AP4M1* promotes proliferation, colony formation, cell migration, and invasion ability of HCC cells in vitro.

## Discussion

In most cases of HCC, patients are diagnosed at an advanced stage and do not have the opportunity to undergo surgical resection. Therefore, reliable biomarkers could help diagnose HCC earlier and accurately predict survival prognosis. In this study, we identified the diagnostic and prognostic value of *AP4M1* in HCC, and the biological function thataffect the development of HCC. In addition, *AP4M1* can be used for the prediction of immune cell infiltration and immune phenotype in hepatocellular carcinoma and positively correlates with various immune checkpoint-related genes, which laid a foundation for future new immunotherapies for HCC.

The expression of *AP4M1* and its potential effect on prognosis in HCC patients have not yet been evaluated. In the present study, we measured that the mRNA level of *AP4M1* was higher in HCC tissues compared to normal tissues in both GEO and TCGA databases. We also found that AP4M1 protein expression was upregulated in HCC tissues compared with normal tissues from the CPTAC database. Furthermore, we discovered that *AP4M1* has a relatively higher ROC score with an AUC of 0.963 in HCC. In addition, we compared *AP4M1* with three other biomarkers. In Suda et al.’s study, Dickkopf-1 (*DKK-1*), a secreted glycoprotein, was reported as a promising biomarker for diagnosing HCC [28]. In Sun Y et al.’s study, Annexin A2 (*AXAN2*), a phospholipid-binding protein, was reported to be involved in the growth and metastasis of HCC, and was also a potential biomarker for HCC [29]. In addition, several studies have revealed Glypican-3 (*GPC3*) as a promising diagnostic biomarker in HCC [30]. Therefore, we analyzed its predictive ability in the TCGA HCC cohort, and showed the ROC curve of AUC values were 0.749, 0.895 and 0.919 respectively. Taken together, *AP4M1* presented a better performance in the diagnosis of HCC patients, and may be applied to further large-scale study in the future. By exploring the correlation between *AP4M1* gene and clinical features, it was found that the overexpression of *AP4M1* was significantly correlated with various clinical features, and a trend toward increased *AP4M1* expression with advanced cancer stages (T3 and T4) and grades (G3 and G4). However, there was no significant difference in *AP4M1* expression between lymph node metastasis and distant metastasis, which may be due to insufficient sample size and the need to increase the number of cases to facilitate future analytical studies. AFP is one of the most widely used biomarker for liver cancer [31]. We also explored differences in *AP4M1* expression among different AFP expression, suggesting that *AP4M1* is able to identify changes in AFP levels and may be used as a candidate biomarker for early diagnosis of HCC. Therefore, we found that *AP4M1* contributes significantly to HCC progression, which aroused our interest to investigate its biological role.

We further analyzed the prognostic impact of *AP4M1* on patients with hepatocellular carcinoma. KM survival curve showed that high *AP4M1* expression was may associated with inferior prognosis in HCC, and patients with high *AP4M1* expression had lower OS and DFS. Univariate and multifactorial COX regression analyses demonstrated that *AP4M1* was an independent risk factor affecting the prognosis of HCC. Thus, our results revealed that *AP4M1* had a predictable effect on clinical features and could serve as a potential prognostic biomarker in HCC.

Specific genetic alterations may promote the tumorigenesis. To investigate whether *AP4M1* mutation played a crucial role in hepatocarcinogenesis, we investigated specific genetic alterations in HCC. The percentage of *AP4M1* genetic alterations in HCC was 11%, and the these genetic alterations presented a significant association with unfavorable OS and DFS. Additionally, our results showed that patients with *AP4M1* high expression levels also displayed higher *TP53* mutation in HCC.

The heterogeneity of the tumor immune microenvironment is an important factor in promoting tumor progression, recurrence and drug resistance. Immune infiltrating immune cells (TIICs) could modulate the process of development as well as the progression of tumors [32]. Studies have shown that a high infiltration of cytotoxic T lymphocytes usually suggested a favorable prognosis for patients, but cytotoxic T lymphocyte deactivation and depletion in hepatocellular carcinoma may cause dysregulation of the tumor microenvironment [33]. In the present study, we observed a significant negative correlation between *AP4M1* and the degree of infiltration of multiple antitumor immune response cells, including CD8^+^ T cells, Th17 cells, DC cells, and pDC cells in HCC by ssGSEA analysis. In recent years, the recommendation of immunotherapeutic strategies including immune checkpoint inhibitors, either as a single agent or in combination with approved local and systemic therapies, has significantly altered the treatment outcome of HCC. Thus, we further analyzed the relationship between *AP4M1* and immune checkpoint-related genes. Our results displayed a significant positive correlation between *AP4M1* and the levels of T-cell failure markers such as PD-1 and CTLA4 in HCC. These markers are key suppressive immune checkpoint proteins that naturally inhibit T-cell activity and allow tumor cells to escape immune surveillance, and playing an important role in maintaining self-tolerance. Meanwhile, the upregulation of these markers enhances the suppressive effect of anti-tumor immunity. Although our observation was preliminary and no study reported the exact effect of *AP4M1* in immune-related processes, we revealed a possible role of *AP4M1* in tumor immune microenvironment, which was proposed to be an in-depth exploration for future investigation.

Furthermore, we validated the impact of *AP4M1* on the ability of proliferation, invasion, and migration of HCCin vitro. We found that the malignant phenotype of HCC cells was suppressed when *AP4M1* knocked down, indicating an oncogenic role of *AP4M1* in HCC. Our study provides a new idea for the molecular function of *AP4M1* and can be further investigated.

Few studies have reported the role of *AP4M1* in tumors. To explore the biological functions of *AP4M1* in HCC, we analyzed the co-expression genes in HCC and performed functional enrichment analysis. Our results revealed that *AP4M1* were associated with Spliceosome, Proteasome, Cell cycle, cell cycle G2/M phase transition, etc. Based on the results of the GSEA-Hallmark signaling pathway enrichment analysis, we found that *AP4M1*-related genes were enriched in the cell proliferation pathway (G2M checkpoint, E2F targets, Myc targets V1). E2F is located downstream of the cell cycle signaling pathway and can regulate the expression of target genes related to the cell cycle process, controlling important processes such as cell proliferation and differentiation [34]. It has been reported that E2F genes have important roles in the mid-cell regulation of a variety of tumors [35, 36]. These results suggest that *AP4M1* may be involved in regulating the malignant proliferation and progression of HCC, which also provides new insights into exploring the mechanism of *AP4M1* in HCC.

Although our study presents an integrative analysis of the prognostic and biological functional values of *AP4M1* in HCC, there are still some limitations. First, some vital clinical information, such as therapeutic modalities, tumor site and other factors, were not available for analysis in the majority of datasets, which need further prospective studies in the future. Second, *AP4M1*-related signaling pathways and downstream regulatory molecules need to be further explored, and more in vivo and in vitro experiments are required to further validate our observations, which will be the direction of our future study. Third, all of the samples used in our study were collected retrospectively, and analyses were conducted using data from public databases. Therefore, a more convincing prospective study is required to confirm our findings and can be our future research direction.

## Conclusion

In this study, we integratively investigated the diagnostic and predictive value of *AP4M1* in HCC. We found that *AP4M1* was highly expressed in HCC and associated with unfavorable prognosis, and was an independent risk factor for HCC prognosis. The oncogenic feature of *AP4M1* was also verified by in vitro experiments. Additionally, we also explored that *AP4M1* was closely related to the immune microenvironment of HCC. Taken together, our study suggests that *AP4M1* may be involved in the malignant progression of HCC, as well as the cancer immune regulation, which provides new insights for the diagnosis and treatment of HCC.

### Electronic Supplementary Material

Below is the link to the electronic supplementary material.

Supplementary Material 1

## Data Availability

The datasets presented in this study can be found in online repositories. The names of the repository/repositories and accession number(s) can be found in the article/**Supplementary Material**.

## Abbreviations

AUC: The Area Under the Curve
BP: Biological processes
BEST: Biomarker Exploration of Solid Tumors
CC: Cellular components
CI: Confidence intervals
DSS: Disease Specific Survival
GSEA: Gene Set Enrichment Analysis
GO: Gene Ontology
HRs: Hazard ratios
HCC: Hepatocellular carcinoma
KM: Kaplan-Meier
KEGG: Kyoto Encyclopedia of Genes and Genomes
MF: Molecular functions
OS: Overall survival
PFS: Progression Free Survival
RFS: Recurrence Free Survival
ssGSEA: Single-sample Gene Set Enrichment Analysis
TCGA: The cancer Genome Atlas
TIMER: The Tumor Immune Estimation Resource
