# Supplementary material for: AP4M1 as a prognostic biomarker associated with cell proliferation, migration and immune regulation in hepatocellular carcinoma
Source: Cancer Cell Int. 2023 Oct 11;23:235. doi: 10.1186/s12935-023-03089-0 (PMC10568912; doi:10.1186/s12935-023-03089-0)
Supplement: Supplementary file 1 — Supplementary Material 1 [file 12935_2023_3089_MOESM1_ESM.docx]

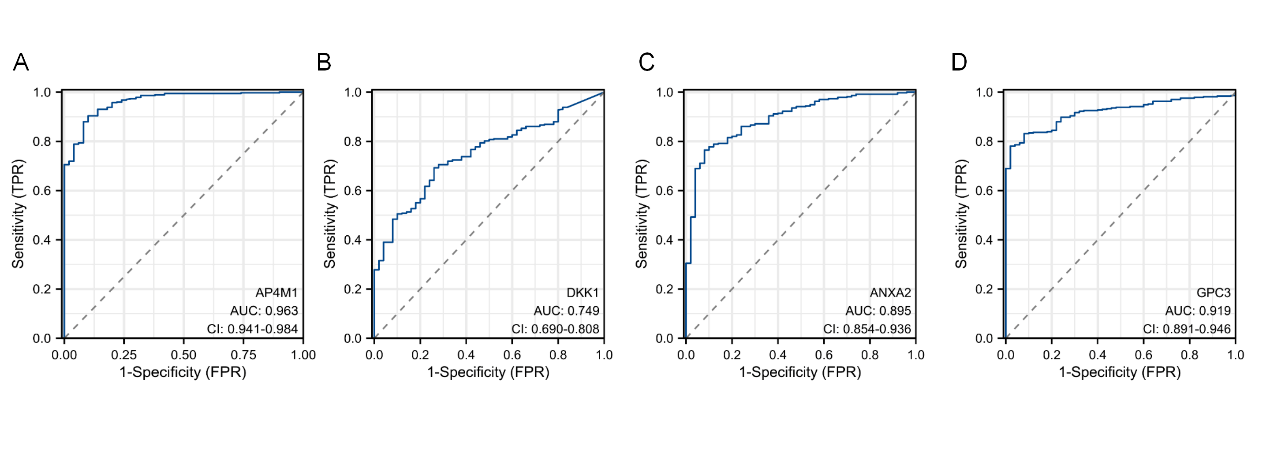


**SUPPLEMENTARY FIGURE 1. The diagnostic ROC curves of *AP4M1* and three other hepatocellular carcinoma biomarker expression levels differentiate HCC tissues from normal tissues in TCGA cohort. (A)** The diagnostic ability of *AP4M1.* **(B)** The diagnostic ability of *DKK1*. **(C)** The diagnostic ability of ANXA2. **(D)** The diagnostic ability of *GPC3*.


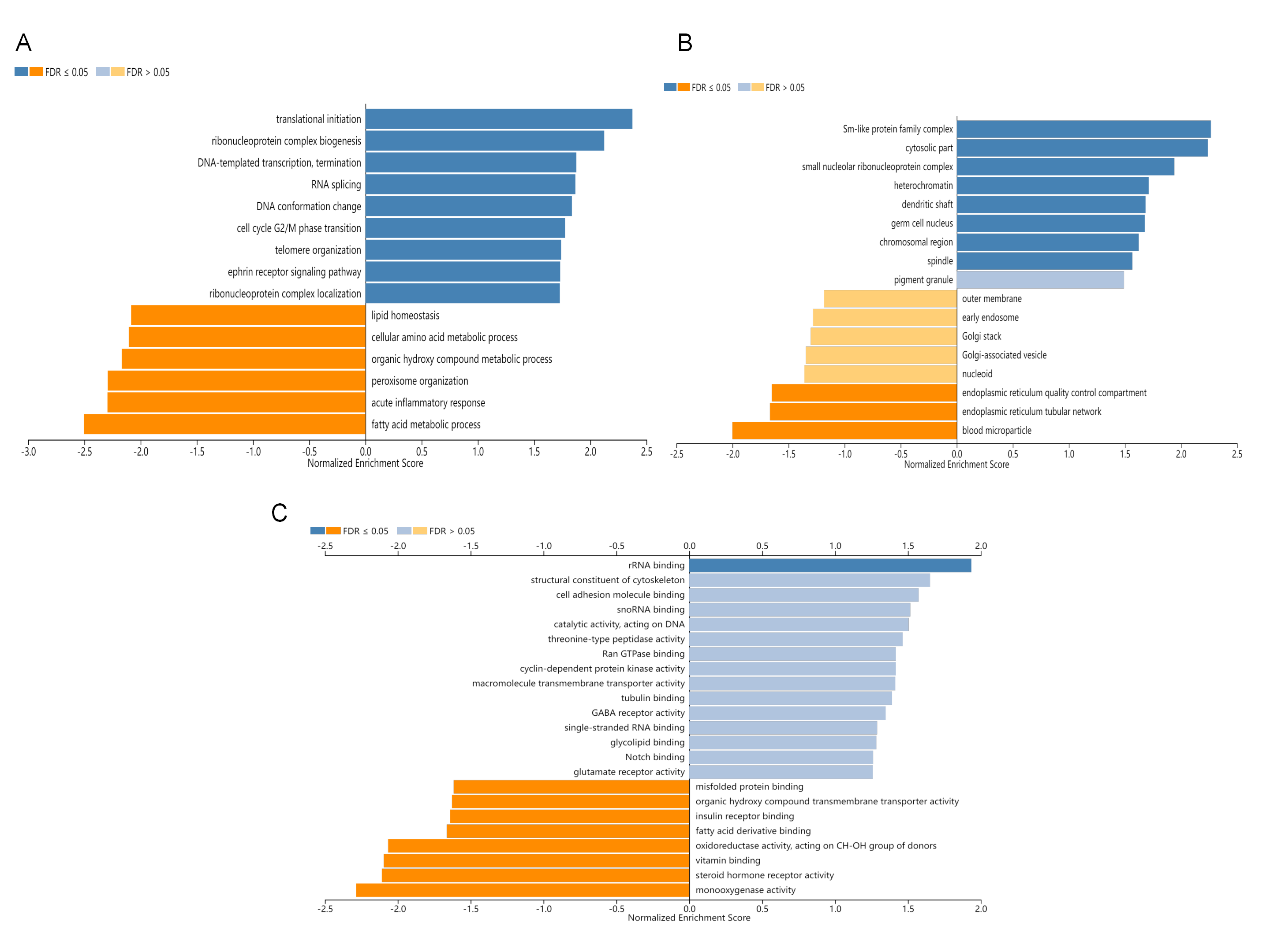


**SUPPLEMENTARY FIGURE 2. GO enrichment analysis based on Linkedomics database (A)**Biological processes analysis **(B)** Cellular components **(C)** Molecular functions.

**Supplementary Table 1.** Clinicopathological characteristics of patients from TCGA cohort.

| Characteristics | Low expression of *AP4M1* | High expression of *AP4M1* | P value |
| --- | --- | --- | --- |
| n | 187(2.9±0.502) | 187(1.872±0.256) |  |
| Age, median (IQR) | 62 (53, 69) | 60 (51, 68) | 0.184 |
| Gender, n (%) |  |  | 0.320 |
| Male | 131 (35%) | 122 (32.6%) |  |
| Female | 56 (15%) | 65 (17.4%) |  |
| Race, n (%) |  |  | 0.408 |
| Asian | 73 (20.2%) | 87 (24%) |  |
| Black or African American | 10 (2.8%) | 7 (1.9%) |  |
| White | 95 (26.2%) | 90 (24.9%) |  |
| BMI, n (%) |  |  | **0.026** |
| <= 25 | 78 (23.1%) | 99 (29.4%) |  |
| > 25 | 90 (26.7%) | 70 (20.8%) |  |
| Weight, n (%) |  |  | **<0.001** |
| <= 70 | 73 (21.1%) | 111 (32.1%) |  |
| > 70 | 101 (29.2%) | 61 (17.6%) |  |
| Tumor status, n (%) |  |  | 0.221 |
| Tumor free | 107 (30.1%) | 95 (26.8%) |  |
| With tumor | 71 (20%) | 82 (23.1%) |  |
| Pathologic stage, n (%) |  |  | **0.030** |
| Stage I | 99 (28.3%) | 74 (21.1%) |  |
| Stage II | 39 (11.1%) | 48 (13.7%) |  |
| Stage III | 33 (9.4%) | 52 (14.9%) |  |
| Stage IV | 3 (0.9%) | 2 (0.6%) |  |
| Histologic grade, n (%) |  |  | **<0.001** |
| G1 | 37 (10%) | 18 (4.9%) |  |
| G2 | 102 (27.6%) | 76 (20.6%) |  |
| G3 | 40 (10.8%) | 84 (22.8%) |  |
| G4 | 5 (1.4%) | 7 (1.9%) |  |
| Pathologic T stage, n (%) |  |  | **0.031** |
| T1 | 105 (28.3%) | 78 (21%) |  |
| T2 | 40 (10.8%) | 55 (14.8%) |  |
| T3 | 33 (8.9%) | 47 (12.7%) |  |
| T4 | 6 (1.6%) | 7 (1.9%) |  |
| AFP(ng/ml), n (%) |  |  | **0.006** |
| <= 400 | 121 (43.2%) | 94 (33.6%) |  |
| > 400 | 24 (8.6%) | 41 (14.6%) |  |
